# Supplementary figures and images for: NF-YA transcription factors suppress jasmonic acid-mediated antiviral defense and facilitate viral infection in rice
Source: PLoS Pathog. 2022 May 13;18(5):e1010548. doi: 10.1371/journal.ppat.1010548 (PMC9132283; doi:10.1371/journal.ppat.1010548)

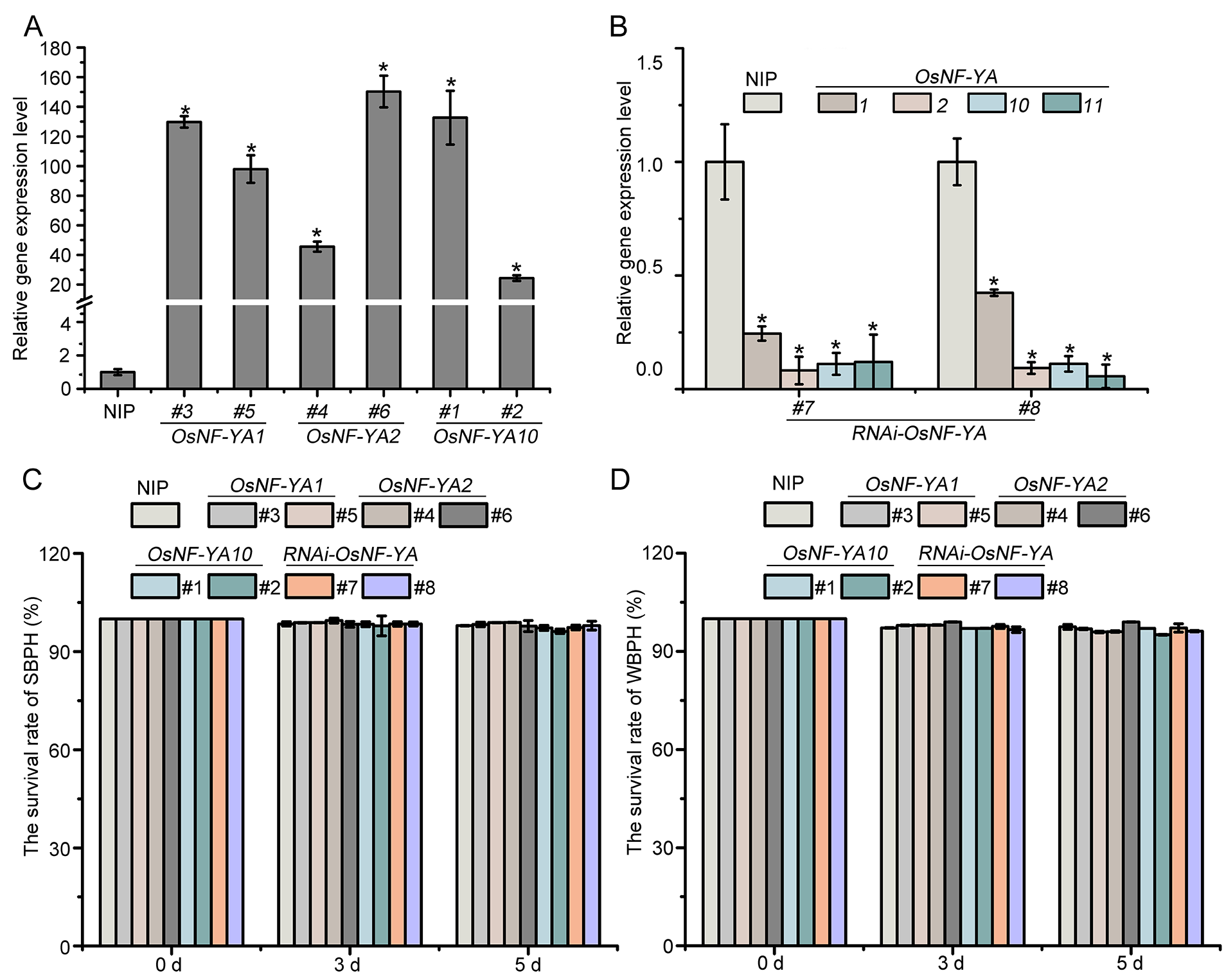

Supplement: S1 Fig — The relative expression levels of OsNF-YAs genes determined in RNA extracted from OE-OsNF-YAs (A) or RNAi-OsNF-YAs (B) plants at 15 dpi. Data are shown as relative expression levels of transgenic plants in comparison with mock plants. C and D. The survival rates of small brown planthoppers (SBPH) and white-backed planthoppers (WBPH) on NIP and OsNF-YAs transgenic plants. Ten-day-old seedlings of OsNF-YAs transgenic lines were infested with virus-free SBPH (C) and WBPH (D). 3 insects were placed on each seedling for 5 days and the numbers surviving were counted 3 and 5 days later. (TIF) [file ppat.1010548.s001.tif]

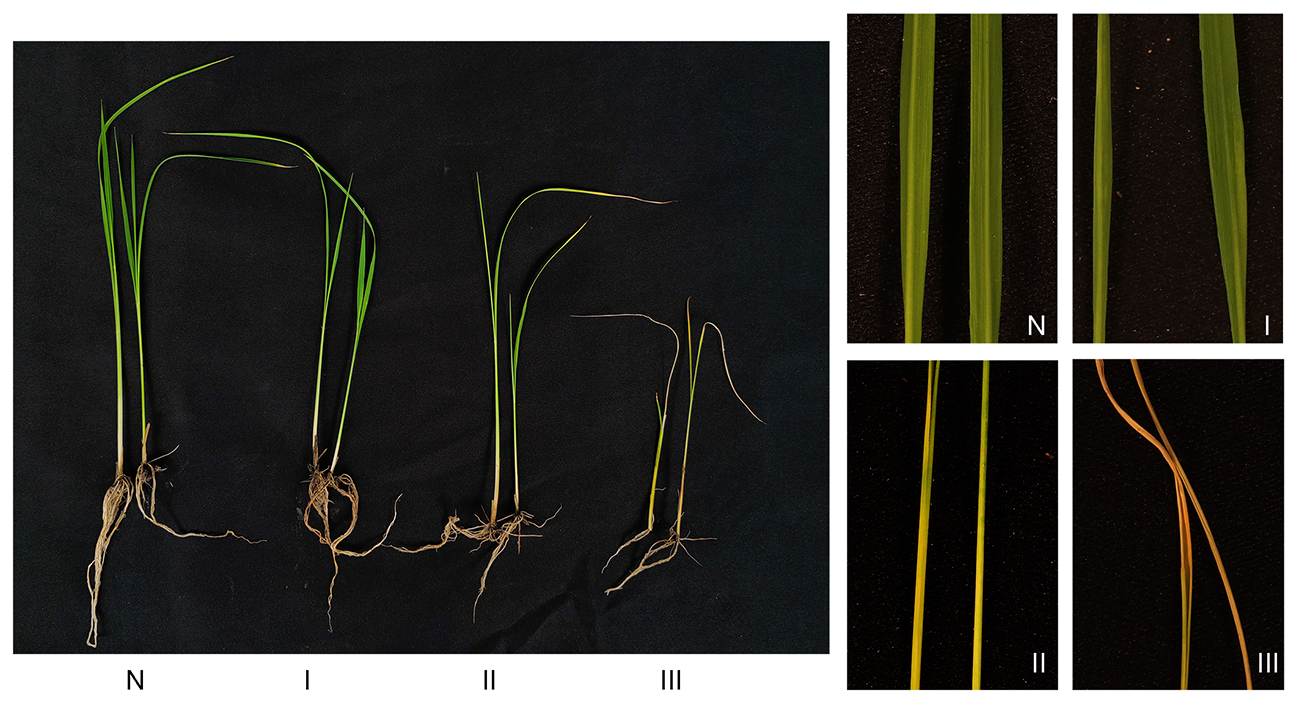

Supplement: S2 Fig — According to the severity of the symptoms, we classified RSV infected plants into four grades: healthy (N), mild mosaic (I), severe mosaic (II), wilting (III). (TIF) [file ppat.1010548.s002.tif]

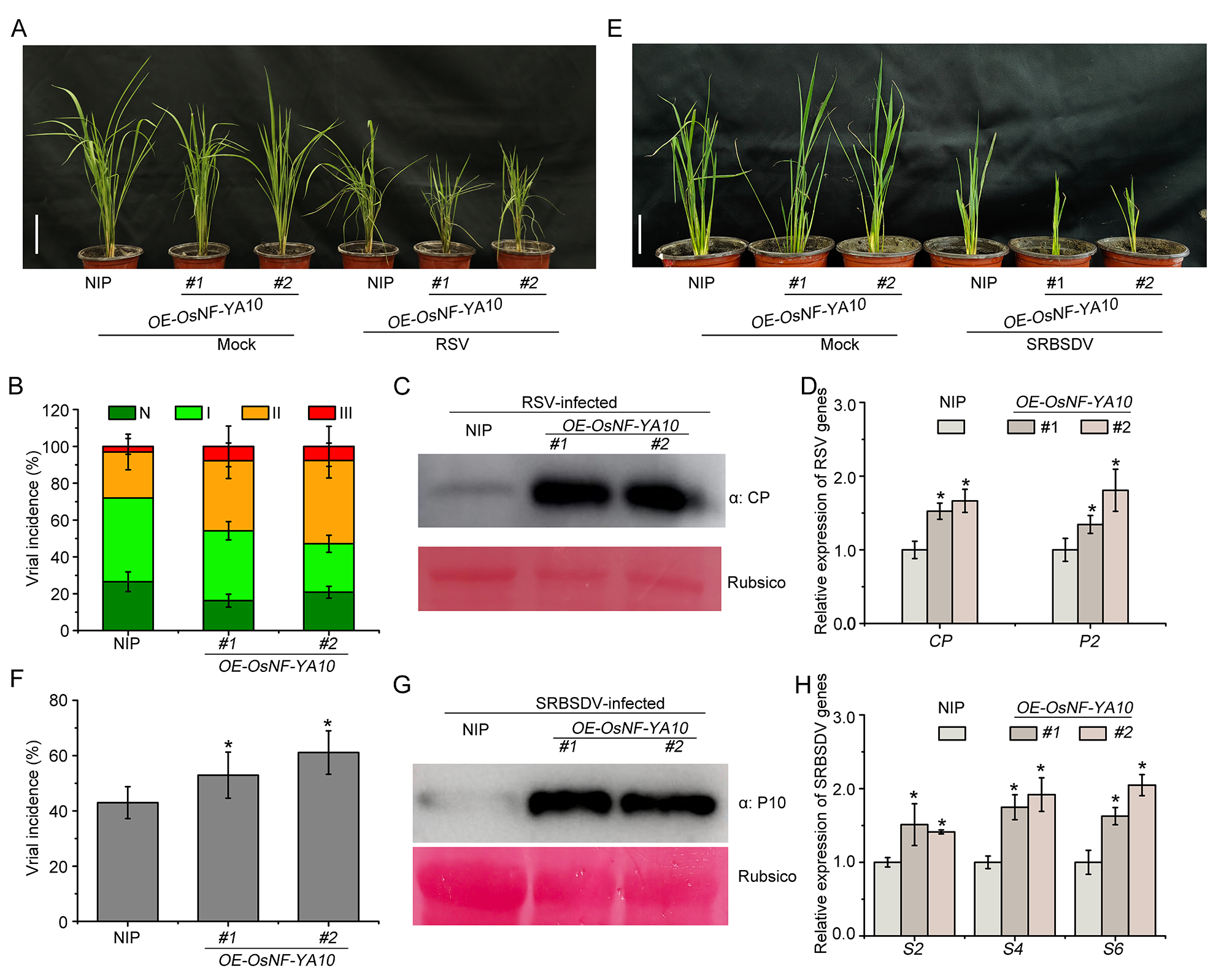

Supplement: S3 Fig — A. The symptoms of RSV infection in OE-NF-YA10 transgenic lines and NIP controls. The phenotypes were observed and photos were taken at 20 dpi. (Scale bars, 5 cm.) B. The percentage of RSV-infected plants with different degrees of disease. N, Healthy; I, mild mosaic; II, severe mosaic; III, wilting. C. The accumulation of RSV CP protein in RSV-infected plants determined by western blotting. Rubsico was used as the internal reference. D. qRT-PCR results showing the relative expression levels of RSV RNAs (CP and P2) in virus-infected plants. E. The symptoms of SRBSDV infection at 30 dpi. (Scale bars, 5 cm.) F. Viral incidence in OE-NF-YAs transgenic lines compared with NIP controls. G. The accumulation of SRBSDV P10 protein in virus-infected plants determined by western blotting. Rubsico was used as the internal reference. H. qRT-PCR results showing the relative expression levels of SRBSDV RNA segments (S2, S4, S6) in virus-infected plants. OsUBQ5 was used as the internal reference gene to normalize the relative expression. Values shown are the means ± SD of 3 biological replicates. Significant differences were identified using Fisher’s least significant difference tests. *At the top of columns indicates significant difference at p ≤ 0.05. (TIF) [file ppat.1010548.s003.tif]

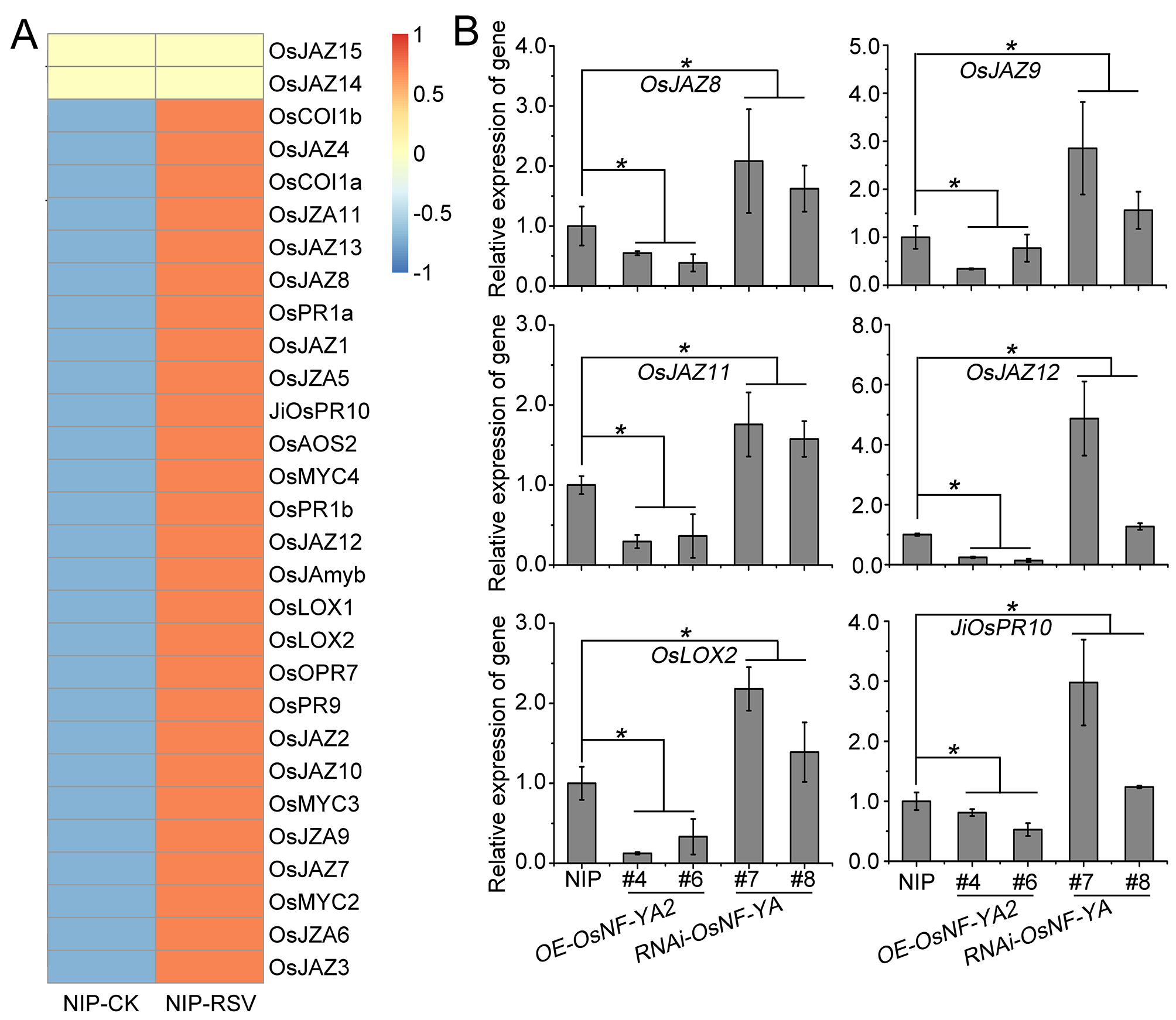

Supplement: S4 Fig — A. Hierarchical clustering showing the changed JA pathway genes in NIP-CK and NIP-RSV. B. qRT-PCR results showing the relative expression levels of JA pathway genes (OsJAZ8; OsJAZ9; OsJAZ11; OsJAZ12; OsLOX2; JiOsPR10) in SRBSDV-infected plants compared with NIP background from three biological replicates in a one-way ANOVA and evaluated at p ≤ 0.05 by Fisher’s least significant difference tests. OsUBQ5 was used as the internal reference gene to normalize the relative expression. Error bars represent SD of 3 biological replicates. Significant differences were identified using Fisher’s least significant difference tests. *At the top of columns indicates significant difference at p ≤ 0.05. (TIF) [file ppat.1010548.s004.tif]

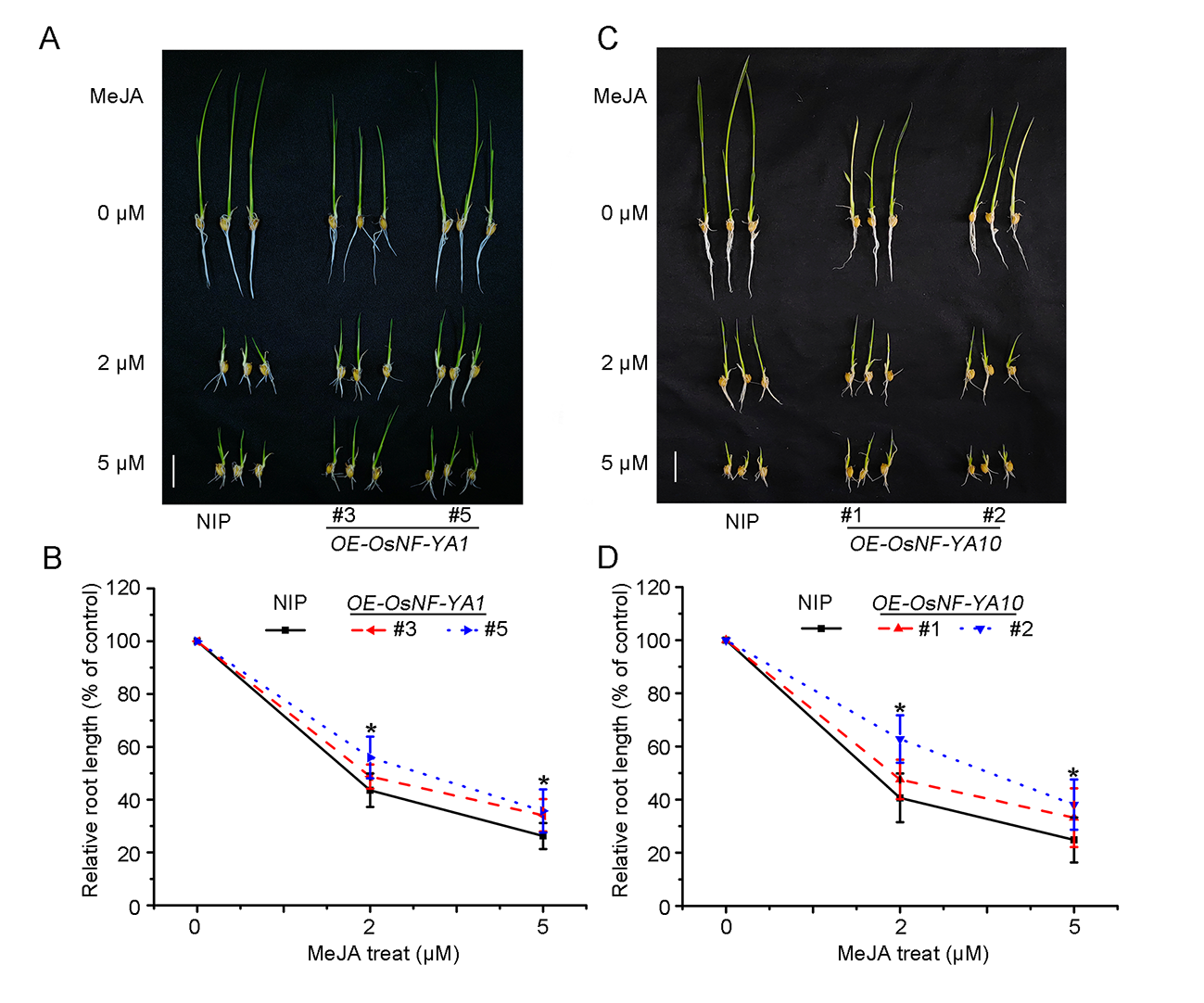

Supplement: S5 Fig — A and C. Phenotypes of OE-OsNF-YA1 (A) and OE-OsNF-YA10 (C) transgenic plants grown on rice nutrient solution containing 2 μM or 5 μM MeJA for 5 days. Scale bar, 2 cm. B and D. Root lengths of OE-OsNF-YA1 (B) and OE-OsNF-YA10 (D) transgenic plants following MeJA treatment. The root lengths of five-day-old seedlings grown in normal rice culture solutions supplemented with different concentrations of MeJA were measured. Data shown are the means from at least 15 seedlings for each sample. Error bars represent SD. Different letters at the top of columns indicate significant difference a p ≤ 0.05 by Fisher’s LSD tests. (TIF) [file ppat.1010548.s005.tif]

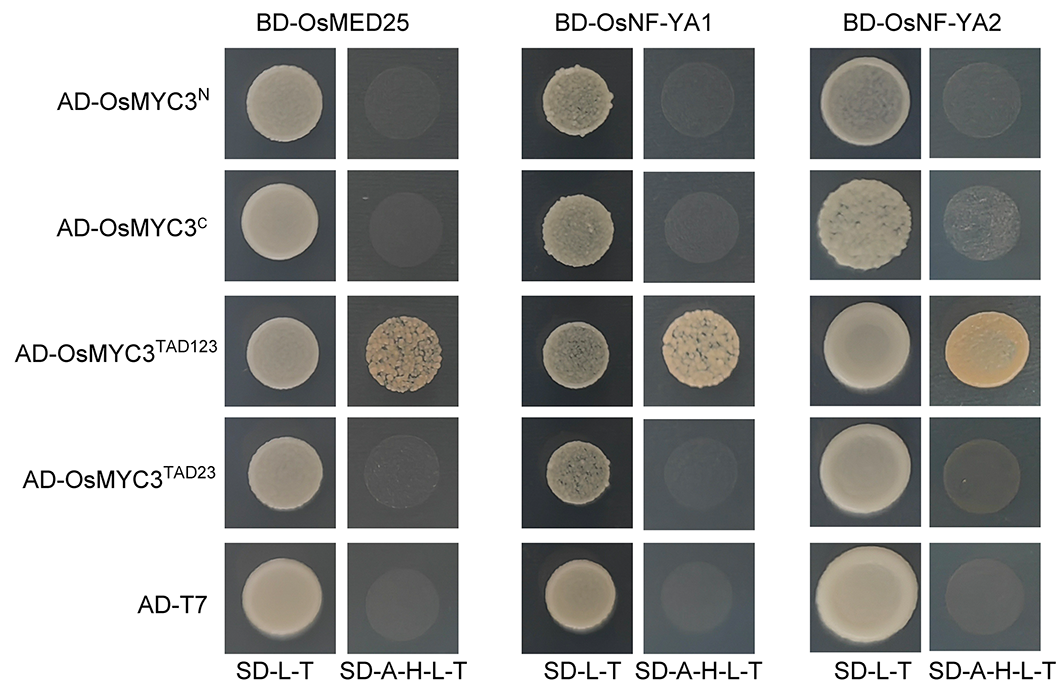

Supplement: S6 Fig — Interaction of OsMED25 and OsMYC3 mutant proteins in a yeast two-hybrid assay. The CDS for OsNF-YAs and OsMED25 were introduced into pGBKT7 and OsMYC3 mutants were introduced into pGADT7 vectors, respectively. Yeast cells co-expressing OsNF-YAs or OsMED25 and OsMYC3 mutants were grown on selective media SD/-Trp/-Leu (SD-L-T), and interactions were tested with SD/-Trp/-Leu/-His/-Ade (SD-L-T-H-A). Pictures were taken after 3 days incubation at 30°C. (TIF) [file ppat.1010548.s006.tif]

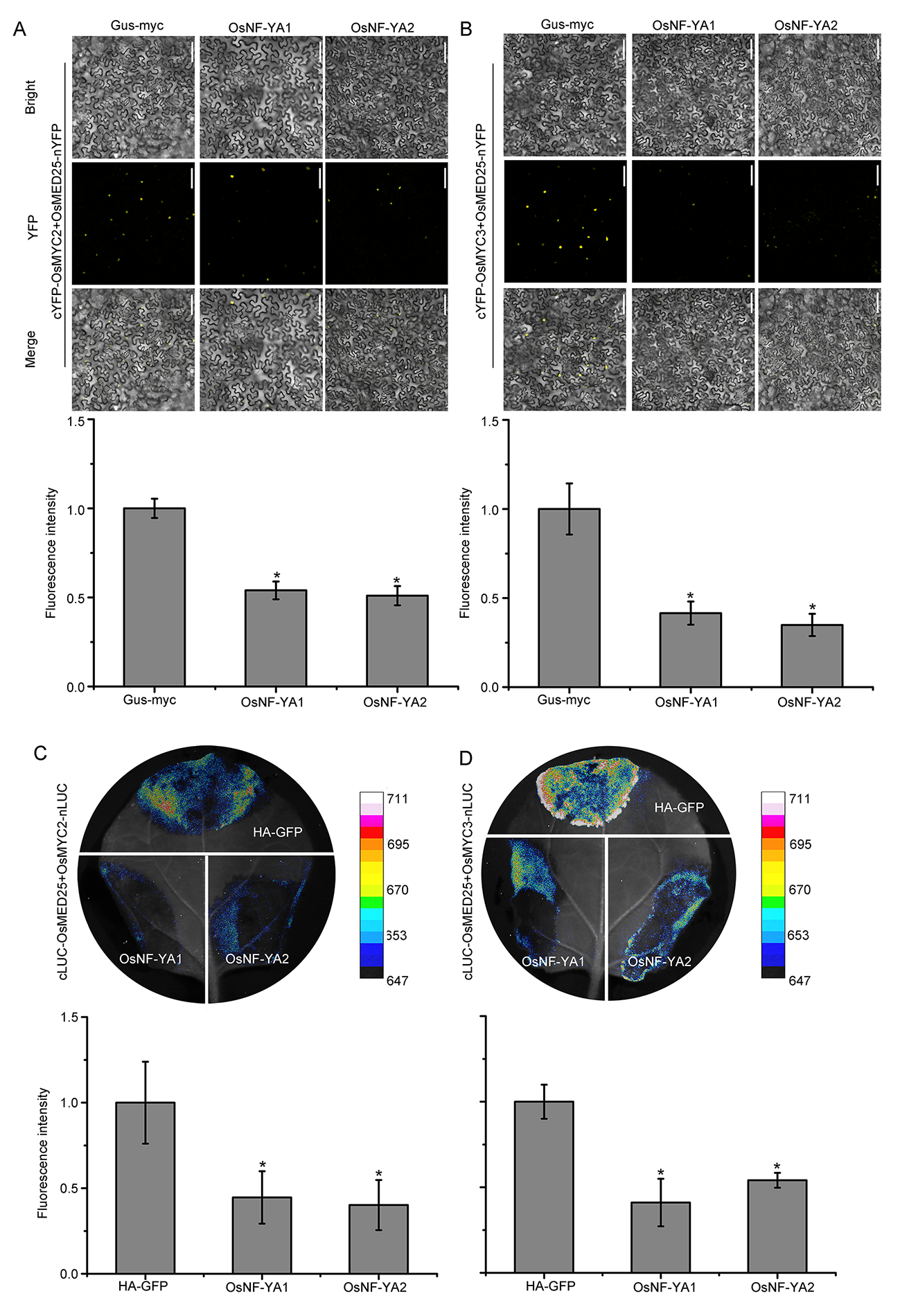

Supplement: S7 Fig — A-B. BiFC assays showing that OsNF-YA1/2 interfere with the interaction between OsMED25 and OsMYC2/OsMYC3. Fusion proteins were transiently expressed in leaves of N. benthamiana and observed by confocal microscopy. The YFP signals were reduced in the presence of OsNF-YA1/2. (Scale bar, 100 μm). Quantification of the fluorescence intensity from leaves co-expressing cYFP-OsMYC2 or cYFP-OsMYC3 and OsMED25-nYFP with Gus-myc or OsNF-YA1/2. Data are means from 40 to 60 transfected cells. Error bars represent SD. Significant differences were identified using Fisher’s least significant difference tests. *At the top of columns indicates significant difference at p ≤ 0.05. C, D. LCI assays showing that OsNF-YA1/2 interferes with the interaction between OsMED25 and OsMYC2/OsMYC3. Fusion proteins were transiently expressed in leaves of N. benthamiana and the measurements of luciferase activity due to LUC reconstitution for the different combinations. Quantification of the fluorescence intensity from leaves co-expressing cLUC-OsMED25 and OsMYC2-nLUC or OsMYC3-nLUC with HA-GFP or OsNF-YA1/2. Error bars represent SD of 3 biological replicates. Significant differences were identified using Fisher’s least significant difference tests. *At the top of columns indicates significant difference at p ≤ 0.05. (TIF) [file ppat.1010548.s007.tif]

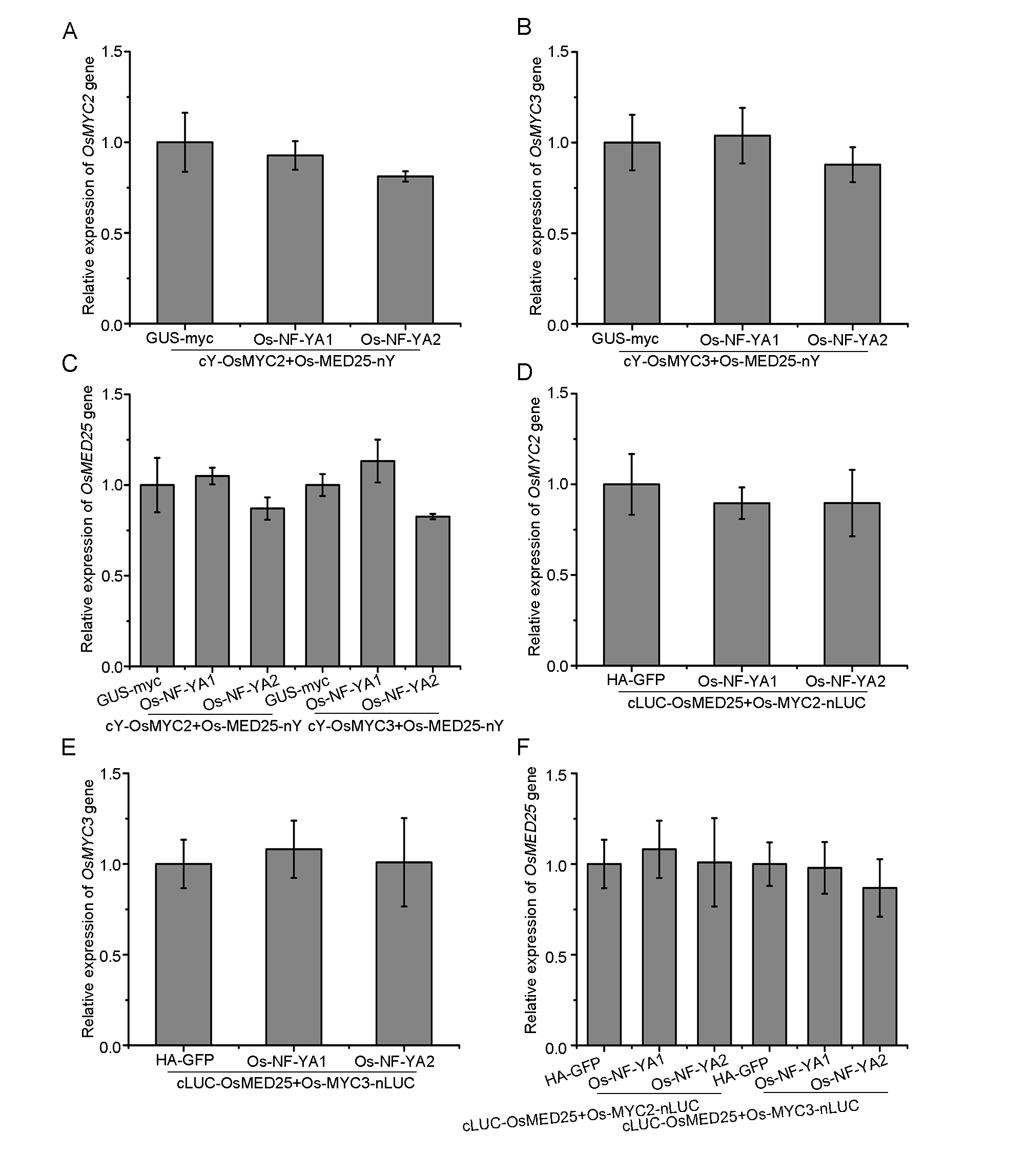

Supplement: S8 Fig — A-F. qRT-PCR results showing the transcript expression of OsMYC2/3 and OsMED25 in these BiFC and LCI assays. The relative expression levels of genes (OsMED25; OsMYC2; OsMYC3) in these BiFC (A-C) and LCI (D-F) assays using three biological replicates in a one-way ANOVA and evaluated at p ≤ 0.05 by Fisher’s least significant difference tests. NbUBC was used as the internal reference gene to normalize the relative expression. Error bars represent SD of 3 biological replicates. (TIF) [file ppat.1010548.s008.tif]

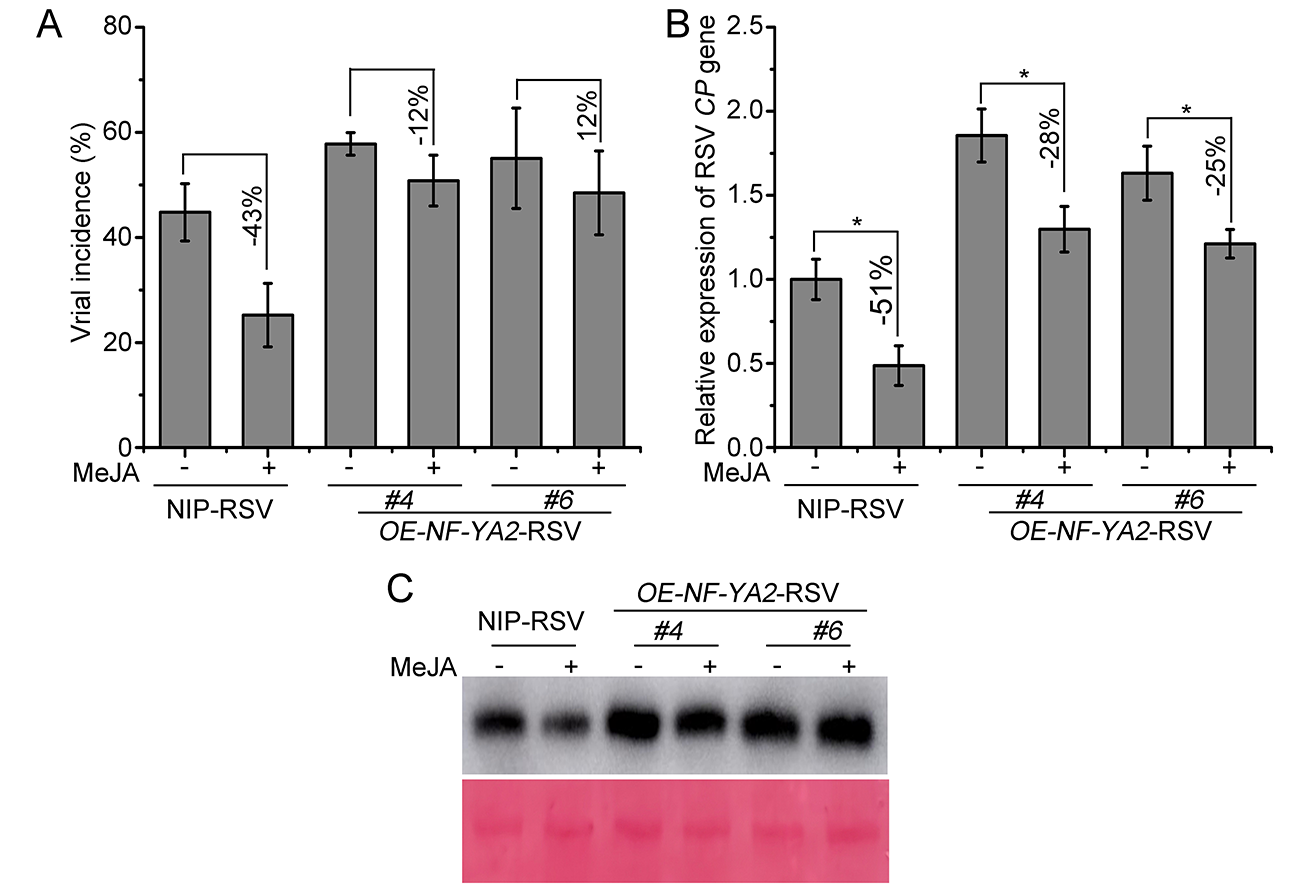

Supplement: S9 Fig — A. Viral incidence in OE-OsNF-YA2 transgenic lines and NIP controls under JA treatment. B. qRT-PCR results showing the relative expression levels of RSV CP gene in virus-infected plants. OsUBQ5 was used as the internal reference gene to normalize the relative expression. Values shown are the means ± SD of 3 biological replicates. Significant differences were identified using Fisher’s least significant difference tests. *At the top of columns indicates significant difference at p ≤ 0.05. C. The accumulation of RSV CP protein in virus-infected plants determined by western blotting. Rubsico was used as the internal reference. (TIF) [file ppat.1010548.s009.tif]

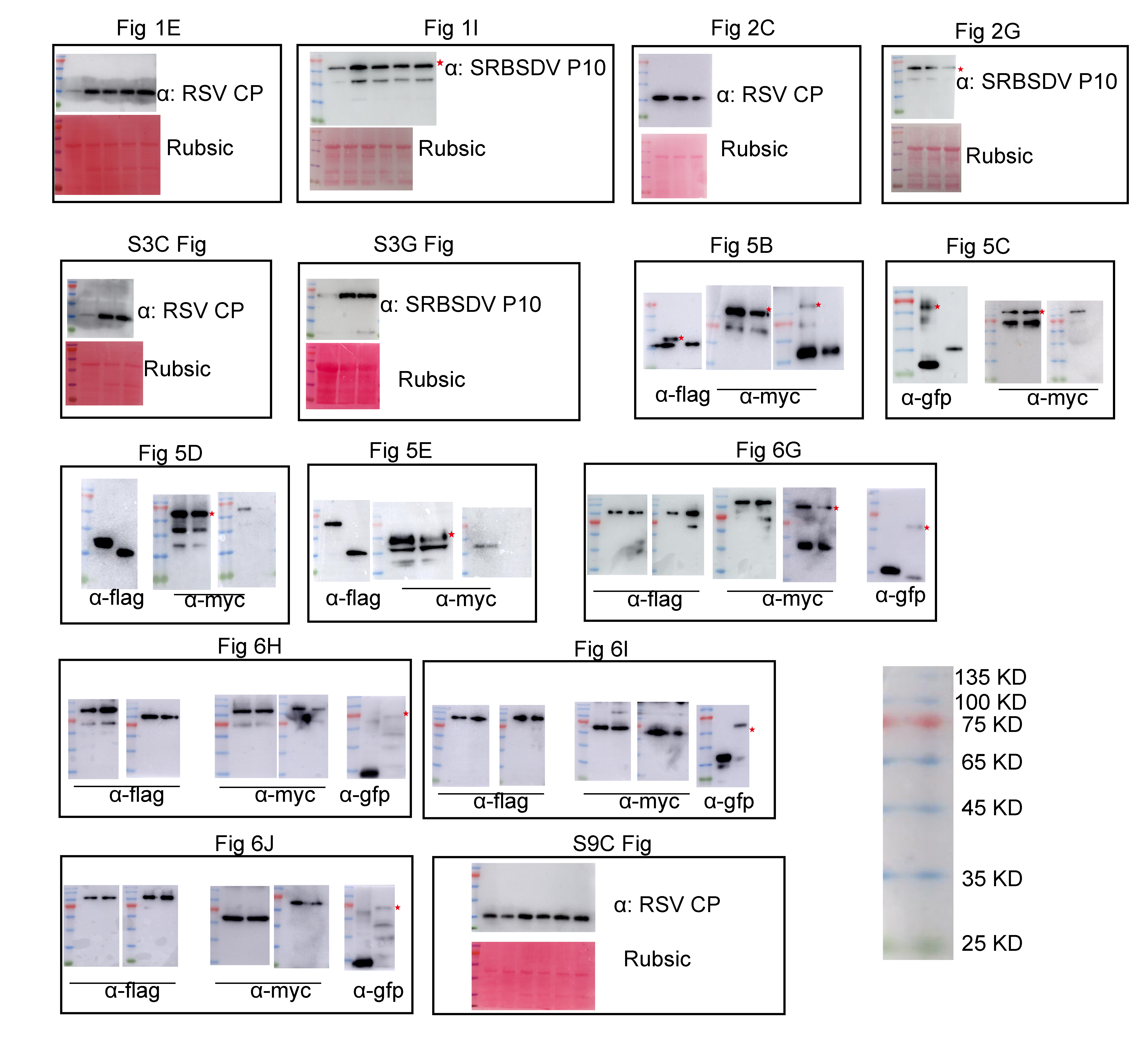

Supplement: S10 Fig — (TIF) [file ppat.1010548.s010.tif]
