# Supplementary material for: NF-YA transcription factors suppress jasmonic acid-mediated antiviral defense and facilitate viral infection in rice
Source: PLoS Pathog. 2022 May 13;18(5):e1010548. doi: 10.1371/journal.ppat.1010548 (PMC9132283; doi:10.1371/journal.ppat.1010548)
Supplement: Attachment — Submitted filename: response letter-3-28 MJA.docx [file ppat.1010548.s016.docx]

**Response Letter**

Reviewer's Responses to Questions

**Part I - Summary**
Please use this section to discuss strengths/weaknesses of study, novelty/significance, general execution and scholarship.

Reviewer #1: Rice viral diseases are the major threats to rice production and food security worldwide. Among them, RSV and SRBSDV are two important viruses in Asia. In this manuscript, Tan et al showed evidence that RSV and SRBSDV infection both induced the expression of OsNF-YA family genes, which inhibit JA mediated antiviral defense by dissociating the OsMYC2/3 - OsMED25 complex to facilitate virus infection. Overall, the story is interesting and novel. However, there are weakness and mistakes that need to be addressed before acceptance for publication.

Reviewer #2: The manuscript of Tan et al., explores the role of rice NF-YA family genes in antiviral defense against two different plant viruses, RSV and SRBSDV. The authors claim that OsNF-YAs physically interact with JA signaling transcription factors OsMYC2/3 and interfere with JA signaling by dissociating the OsMYC2/3-OsMED25 complex, which repress JA-mediated antiviral immunity.
Overall, the manuscript is well-written, the data generated throughout the manuscript are solid and the figure are clear. The conclusions drawn by the authors are supported by the data. This research provides valuable understanding about the interplay between NF-Y transcription factors and JA signaling in the context of plant resistance against virus in rice.

Reviewer #3: Dear Editor,
The author investigated the role of NF-Y transcription factors during viral infection with Rice stripe virus (RSV) and Southern rice black-streaked dwarf virus (SRBSDV). The author show that the virus induced overexpression of certain NF-Y genes is important for viral pathogenicity. Further experiments showed that OsNF-YAs physically interact with JA signaling transcription factors OsMYC2/3 and interfere with JA signaling by dissociating the OsMYC2/3-OsMED25 complex, which inhibits the transcriptional activation activity of OsMYC2/3. The results show that OsNF-YAs broadly inhibit plant antiviral defense by repressing JA signaling pathways. This provides new insight into how OsNF-YAs are directly associated with the JA pathway.
In general, the manuscript is well written and the conclusions are well supported by experimental evidences. The authors used different methods to support their results which makes them confident. To my opinion, these results are of high interest for the scientific community as it nicely shows how plant virus infection disturb the JA-pathway. Furthermore, it is a nice follow up of a previous study from these group.

Response: We are very pleased to receive the reviewers’ helpful and positive comments. We have carefully taken these suggestions into consideration in preparing this revision, and have performed additional experiments to address the reviewers’ concerns. Comments received are shown in black below, with our response in red font.

**Part II – Major Issues: Key Experiments Required for Acceptance**
Please use this section to detail the key new experiments or modifications of existing experiments that should be absolutely required to validate study conclusions.
Generally, there should be no more than 3 such required experiments or major modifications for a "Major Revision" recommendation. If more than 3 experiments are necessary to validate the study conclusions, then you are encouraged to recommend "Reject".

Reviewer #1: Major:
1) The authors claim that OsNF-YAs inhibit the transcriptional activation of OsMYC2/3 by direct association with the TAD domain, but the evidence is not convincing. Firstly, Fig6G - Fig6J can`t illustrate the OsNF-YA disrupt the interaction of OsMYCs and OsMED25 through TAD domain, the MYCTAD should be added to the experiment system; Secondly, BiFC and LCI assays are both imaging assays, quantitative analysis should be carried out to directly prove their interaction is disrupted. Besides, the authors should further confirm this conclusion in corresponding transgenic materials.

Response: We are grateful for the reviewer’s suggestions. We have added the Y2H assays, and the results showed that the TAD motif of OsMYC3 is required for interaction with both OsMED25 and OsNF-YA1/2 (Fig 1 below; Fig 6A-6B and S6 Fig in revised manuscript). We then designed the competitive Co-IP and BIFC experiments to elucidate the effect of OsNF-YA1/2 on the OsMYCs-OsMED25 interaction. The results showed that OsNF-YA1/2 compete with OsMED25 to bind OsMYCs (Fig 2 below; Fig 6G-6J in revised manuscript). In addition, quantitative analysis of BIFC and LCI has been added in the revised manuscript (S7 Fig and S8 Fig). Given that OsMED25 is essential for OsMYC2/3 transcriptional activation by binding the TAD domain, we conclude that OsNF-YA1/2 affect OsMYC2/3 transcriptional activation mainly by disrupting the association of and OsMYCs-OsMED25.

Fig 1. The TAD motif of OsMYC3 is required for interaction with OsMED25 or OsNF-YAs.

Fig 2. Competitively Co-IP assays confirm that OsNF-YA1/2 interfere with the OsMYC2/3-OsMED25 interaction.

2) Both RSV and SRBSDV infection induce the expression of the 11 OsNF-YAs, and the authors constructed the overexpression lines of OsNF-YA1, OsNF-YA2 and OsNF-YA10, what`s the reason for choosing OE-OsNF-YA2 lines to perform RNA-seq and JA sensitivity assays?

Response: We first obtained homozygous *OsNF-YA2* transgenic plants and then used them for JA sensitivity and RNA-seq. In the revised manuscript, we have added JA sensitivity assays with both *OsNF-YA1* and *OsNF-YA10* transgenic lines. As expected, similar to *OsNF-YA2*, overexpression of *OsNF-YA1* and *OsNF-YA10* makes rice insensitive to JA treatment (see Fig 3 below and Fig S5 in the revised manuscript).

Fig 3. JA sensitivity assays with *OsNF-YA1* and *OsNF-YA10* transgenic lines.

Reviewer #2: I think this manuscript is suitable for publishing in PloS Pathogens with minor revised. The major concern is below:
They examined the effects of OsNF-YA2 on JA sensitivity by exogenous application of methyl jasmonate (MeJA). They found that overexpression of OsNF-YA2 makes rice plants less sensitive to JA treatment whereas repression of OsNF-YA2 increases sensitivity by measuring the root length. I think they should test the influence of JA on the RSV and SRBSDV resistance of OsNF-YA2 overexpressing and RNAi plants to support they conclusion that NF-YA transcription factors suppress JA signal to facilitate viral infection.

**Response:** Thanks for the reviewer’s suggestions. In the revised manuscript, Exogenous JA was applied to RSV-infected *OE-OsNF-YA2* transgenic plants to evaluate the impact of NF-YAs on JA-mediated antiviral defense. RSV-inoculated seedlings were immediately sprayed with MeJA (50 μM) or dilute Triton X-100 (0.1% Triton) as controls and then grown in a glasshouse. After about 20 days, the viral incidence and viral content were determined. As shown in Fig 4 below and Fig S9 in the revised manuscript, JA treatment significantly enhanced the resistance of wild-type rice plants to RSV infection compared to that of *OE-OsNF-YA2* transgenic plants (43% VS 12%). qRT-PCR and Western blotting experiments confirmed that JA treatment significantly reduced the accumulation of RSV CP level in wild-type rice plants (51%), while the effect of JA treatment was much alleviated in *OE-OsNF-YA2* transgenic plants (28% or 25%) (S9B Fig and S9C Fig). These results indicated that OsNF-YAs make rice insensitive to JA treatment and inhibit the JA-mediated antiviral defense.

**Fig 4. Effects of exogenous JA on RSV-infected *OE-OsNF-YA2* transgenic plants.** A. Viral incidence in *OE-NF-YA2* transgenic lines and NIP controls under JA treatment. B. qRT-PCR results showing the relative expression levels of RSV *CP* gene in virus-infected plants. *OsUBQ5* was used as the internal reference gene to normalize the relative expression. Values shown are the means ± SD of 3 biological replicates. Significant differences were identified using Fisher’s least significant difference tests. *At the top of columns indicates significant difference at p ≤ 0.05. C. The accumulation of RSV CP protein in virus-infected plants determined by western blotting. Rubsico was used as the internal reference.

Reviewer #3: Figure 6G: Have you infiltrated a culture with an empty plasmid in the variant cYFP-OsMYC2+OsMED25-n-YFP in order to account for differences in the number of infiltrated cultures compared to the other treatments where OsNF-YA1/2? Maybe it would be good to co-infiltrate a OsNF-YA1/2 mutants unable to interact with OsMYC2/3 as control.

**Response:** Thanks for the reviewer’s suggestions. Currently, we haven’t found the suitable mutants of OsNF-YA1/2 for these BIFC assays, but we have added Gus-myc as the control in the revised manuscript (S7A and S7B Fig). The transcript expression of cYFP-OsMYC2 and OsMED25-nYFP showed no obvious difference in these BIFC samples (S8A - S8C Fig), indicating that the decreased fluorescence intensity of cYFP-OsMYC2 and OsMED25-nYFP in the presence of OsNF-YA1/2 was not caused by transcript expression differences. To further verify the influence of OsNF-YA1/2 on the OsMYC2-OsMED25 interaction, we have added Co-IP assays to confirm our conclusion that OsNF-YA1/2 really interferes with the OsMYCs-OsMED25 interaction (see Fig.5 below and Fig.6G-6J in the revised manuscript).

Fig 5. The competitive Co-IP assays to confirm that OsNF-YA1/2 interfere with the OsMYCs-OsMED25 interaction

**Part III – Minor Issues: Editorial and Data Presentation Modifications**
Please use this section for editorial suggestions as well as relatively minor modifications of existing data that would enhance clarity.

Reviewer #1: Minor:
3) The authors concentrate solely on the expression level of OsNF-YAs after virus infection and the subsequent experiments, since OsNF-Y is a heterotrimeric complex consisting of OsNF-YA, OsNF-YB and OsNF-YC subunit, did OsNF-YB and OsNF-YC also play roles in antiviral defense? How do they respond to virus infection? Do they interact with OsMYC2/3?

**Response:** Following the reviewer’s suggestions, we have performed qRT-PCR assays to test the relative expression level of *OsNF-YB* and *OsNF-YC* family genes in response to virus infection. As shown in Fig 6 below, In contrast to the increased transcript expression of *OsNF-YAs*, the expression of most of *OsNF-YB* and *OsNF-YC* family genes was significantly decreased after RSV or SRBSDV infection, indicating that *OsNF-Y* family genes might be differentially involved in plant defense in response to virus infection. In this study, we mainly focus on the roles of *OsNF-YAs* in rice antiviral defense, and the roles of *OsNF-YBs* and *OsNF-YCs* in plant defense will be the subject of future work.

Fig 6. The relative expression level of *OsNF-YB* and *OsNF-YC* family genes after virus infection. A, B. The relative expression levels of *OsNF-YB* family genes after infection by (A) RSV and (B) SRBSDV. RNA was extracted from infected plants at 30 days post inoculation (dpi). Data are shown as relative expression level of *OsNF-YB* family genes in infected plants in comparison with mock plants. C, D. The relative expression levels of *OsNF-YC* family genes after infection by (C) RSV and (D) SRBSDV. RNA was extracted from infected plants at 30 days post inoculation (dpi). Data are shown as relative expression level of *OsNF-Y* family genes in infected plants in comparison with mock plants.

4) The authors find OsNF-YAs negatively regulate JA pathway by qRT-PCR and JA sensitivity assays indirectly, it would be better to measure the JA contents in OE-OsNF-YA lines or Os-NF-YA RNAi mutant lines.

**Response:** Thanks for the reviewer’s suggestions. We have measured the JA contents in various *OsNF-YA* transgenc plants. The results indicated that the jasmonic acid (JA) contents in *OE-OsNF-YA* and *RNAi-OsNF-YA* lines were inconsistent with the JA sensitivity in those transgenic plants (Fig 7 below), suggesting that OsNF-YAs mainly affect the JA signaling pathway rather than the JA biosynthesis pathway.

Fig 7. The jasmonic acid (JA) contents in *OE-OsNF-YA* and *RNAi-OsNF-YA* lines in rice leaves. Values are means SD of three biological replicates.

5) In the yeast two hybrid assay in Fig6A, MYC3N also obtain a TAD domain, it is strange that MYC3N can`t interact with OsNF-YA1 since MYC3TAD123 can interact with OsNF-YA1. Also, it would be better to label the schematic diagram in the left.

**Response:** We made a mistake in the schematic diagram. MYC3N contains an incomplete TAD domain. We have corrected the schematic diagram in Fig 6A of the revised manuscript.

6) I don`t think the photograph of Grade II in FigS2 shows the typical symptoms of RSV infected plants.

**Response:** We now provide a better picture of the viral symptoms in S2 Fig.

7) In the dual-LUC reporter system in Fig6B, the authors use BD empty vector as control, they should choose a better control which can express a certain protein instead of an empty vector.

**Response:** BD-Ven (BD domain fused to fluorescent protein Venus) was used as the control in the dual-LUC reporter system. We have corrected this mistake in the revised manuscript (Fig 6B).

8) The authors use BiFC and LCI assays to show OsNF-YAs can imped the interaction between MYC2/3 and MED25 in Fig6.G-J. Nevertheless, they should carry out western blotting assay to exclude the effect of protein expression level.

**Response:** Due to lack the suitable antibody, we used qRT-PCR to test the transcript expression of MYC2/3 and MED25 in these BiFC and LCI assays. The results showed that the transcript expression of cYFP-OsMYC2 or OsMED25-nYFP showed no obvious difference in these samples (S8A – S8F Fig), indicating that the decreased fluorescence intensity of OsMYC2 and OsMED25 in the presence of OsNF-YA1/2 was not caused by transcript expression differences.

9) Ponceau S staining in Fig1E shows the loading is not equal, which makes this result unreliable.

**Response:** We have corrected this in the revised manuscript (Fig 1E).

10) There are no Y-axis titles and significant analysis in FigS1A and FigS1B.

**Response:** Corrected.

11) The Rubsico in FigS3 is barely visible.

**Response:** We have provided a better figure in the revised manuscript (S3 Fig).

12) Line 316: “This was further confirmed a split luciferase” should be “…confirmed by…”.

**Response:** Corrected (Line 325).

13) Line 317: The full name of LCI assay is luciferase complementation imaging assay.

**Response:** Corrected (Line 326).

14) The authors should scrupulously check the italics in the manuscript because there are some mistakes in writing the name of genes, the overexpression lines and mutants. For example, in line 374 - line 382, the name of genes should be italicized.

**Response:** Corrected (Line 395 - Line 403).

15) Line 882: Rice stripe virus or Southern rice black-streaked dwarf virus should be Rice stripe virus and Southern rice black-streaked dwarf virus.

**Response:** Corrected (Line 1002).

16) Line 888: “The phenotypes were observed and photos taken at 20dpi” should be “…photos were taken at 20dpi”. This mistake also occurs in many other places in the manuscript.

**Response:** Corrected (Line 675, Line 1009, Line 1017, Line 1032, Line 1040).

17) Line 902: UBQ5 should be italicized as it is a gene name. This mistake also occurs in many other places in the manuscript.

**Response:** Corrected (Line 536, 688, 702, 1023, 1072).

18) There is no scale bar in Fig2E.

**Response:** Corrected (Fig 2E).

19) The overexpression line OE-OsNF-YA2 should be italicized in Fig3A.

**Response:** Corrected (Fig 3A).

20) In Fig4 and FigS4, does the gene JiOsJPR10 means jasmonate inducible rice PR10? If so, it should be JiOsPR10 instead of JiOsJPR10.

**Response:** Corrected (Fig 4 and S4 Fig).

21) Line 964: the authors claim the scale bar is 5cm in Fig4C, however, Fig4C is a statistical result, there is no scale bar at all.

**Response:** The scale bar belongs to Fig 4B; we have corrected this in revised manuscript.

22) Line 967: OsNF-YAs and OsMYCs should be italicized type.

**Response:** Corrected (Line 1090, Line 1092).

23) Line 999: Authors claim the scale bar of the microscope photos is 50cm, it should be 50μm.

**Response:** Corrected (Line 1122).

24) Fig6C – Fig6F should be marked * means p≤0.05.

**Response:** We have added this in the revised manuscript (Line 1139 - Line 1140).

**Reviewer #2:** (No Response)

**Reviewer #3:** In principle, I suggest a minor revision with a few comments from my site:
Line 64: Wrong punctuation
**Response:** Corrected (Line 63).

Figure 1J: It must be “genes” on y-axis
**Response:** We have corrected this in the revised manuscript (Fig 1J).

Have the authors an idea whether the higher expression of OsNF-YAs is induced by specific viral protein?
**Response:** This is a very interesting question. We've thought about this question before, and we also tested the expression of OsNF-YAs in RSV *P2* or SRBSDV *P8* transgenic plants, which we had constructed in our previous work. The results showed that the expression of *OsNF-YAs* was not affected in the presence of RSV P2 or SRBSDV P8 in rice plants (see Fig 8 below). RSV or SRBSDV encode several viral proteins and therefore it cannot be ruled out that other viral proteins directly promote the expression of *OsNF-YAs*. It will be worth exploring the expression regulation of *OsNF-YAs* in response to viral infection in the future.

Fig 8. The relative expression level of *OsNF-YAs* in *P2-ox* (A) and *SP8-ox* (B).

How does the author explain the lower/higher viral incidence in transgenic plants? Is an effect on the vector possible?

**Response:** Thanks for the reviewer’s comments. In the revised manuscript, we have tested the effect of various *OsNF-YAs* transgenic plants on the survival of the vector. The results showed that the survival rate of planthoppers did not differ among these plants (S1C and S1D Fig). In viral inoculation experiments, 150 viruliferous planthoppers feed on 30 rice seedlings for 5 days. Usually, these 30 rice plants are not all infected successfully by rice viruses, and the viral incidence is used as an indicator for evaluating plant antiviral ability [1-3].

References:

1.	Yang Z, Huang Y, Yang J, Yao S, Zhao K, Wang D, et al. Jasmonate Signaling Enhances RNA Silencing and Antiviral Defense in Rice. Cell Host & Microbe. 2020;28(1):89-103.e8. <https://doi.org/10.1016/j.chom.2020.05.001>. PubMed PMID: 32504578.

2.	Zhang H, Li L, He Y, Qin Q, Chen C, Wei Z, et al. Distinct modes of manipulation of rice auxin response factor OsARF17 by different plant RNA viruses for infection. Proc Natl Acad Sci. 2020;117(16):9112-21. <https://doi.org/10.1073/pnas.1918254117>. PubMed PMID: 32253321

3.	Li L, Zhang H, Chen C, Huang H, Tan X, Wei Z, et al. A class of independently evolved transcriptional repressors in plant RNA viruses facilitates viral infection and vector feeding. Proc Natl Acad Sci. 2021;118(11):e2016673118. <https://doi.org/10.1073/pnas.2016673118>. PubMed PMID: 33836579.
